# A New Generation of Crystallographic Validation Tools for the Protein Data Bank

**DOI:** 10.1016/j.str.2011.08.006

**Published:** 2011-10-12

**Authors:** Randy J. Read, Paul D. Adams, W. Bryan Arendall, Axel T. Brunger, Paul Emsley, Robbie P. Joosten, Gerard J. Kleywegt, Eugene B. Krissinel, Thomas Lütteke, Zbyszek Otwinowski, Anastassis Perrakis, Jane S. Richardson, William H. Sheffler, Janet L. Smith, Ian J. Tickle, Gert Vriend, Peter H. Zwart

**Affiliations:** 1 CIMR, University of Cambridge, Cambridge CB2 0XY, UK; 2 Lawrence Berkeley Laboratory, Berkeley, CA 94720-8235, USA; 3 Department of Biochemistry, Duke University, Durham, NC 27710, USA; 4 Howard Hughes Medical Institute and Departments of Molecular and Cellular Physiology, Neurology and Neurological Sciences, Structural Biology, and Photon Science, Stanford University, James H. Clark Center, Stanford, CA 94305-5432, USA; 5 Department of Biochemistry, University of Oxford, Oxford OX1 3QU, UK; 6 CMBI, NCLMS, Radboud University Nijmegen Medical Centre, 6525 GA Nijmegen, The Netherlands; 7 Department of Biochemistry, NKI, 1066 CX Amsterdam, The Netherlands; 8 Department of Cell and Molecular Biology, Uppsala University, Biomedical Centre, SE-751 24 Uppsala, Sweden; 9 European Bioinformatics Institute, Hinxton, Cambridge CB10 1SD, UK; 10 STFC Rutherford Appleton Laboratory, Chilton, Didcot OX11 0QX, UK; 11 Justus-Liebig University Gießen, Institute of Veterinary Physiology and Biochemistry, 35392 Gießen, Germany; 12 UT Southwestern Medical Center, Dallas, TX 75390-8816, USA; 13 Department of Biochemistry, University of Washington, Seattle, WA 98195, USA; 14 Life Sciences Institute, Department of Biological Chemistry, University of Michigan, Ann Arbor, MI 48109, USA; 15 Astex Therapeutics, Cambridge CB4 0QA, UK

## Abstract

This report presents the conclusions of the X-ray Validation Task Force of the worldwide Protein Data Bank (PDB). The PDB has expanded massively since current criteria for validation of deposited structures were adopted, allowing a much more sophisticated understanding of all the components of macromolecular crystals. The size of the PDB creates new opportunities to validate structures by comparison with the existing database, and the now-mandatory deposition of structure factors creates new opportunities to validate the underlying diffraction data. These developments highlighted the need for a new assessment of validation criteria. The Task Force recommends that a small set of validation data be presented in an easily understood format, relative to both the full PDB and the applicable resolution class, with greater detail available to interested users. Most importantly, we recommend that referees and editors judging the quality of structural experiments have access to a concise summary of well-established quality indicators.

## Highlights

► Validation criteria used by the PDB for X-ray crystal structures have been reassessed ► Key scores should be presented prominently in an easily understood format ► A concise validation report should be available to referees of papers on crystal structures

## Introduction

Validation arose as a major issue in the structural biology community when it became apparent that some published structures contained serious errors (Brändén and Jones, 1990). In response, the community developed a number of validation criteria, and tools to assess these criteria were implemented by the Protein Data Bank (PDB) (Bernstein et al., 1977; Berman et al., 2000), which later expanded to become the Worldwide PDB (wwPDB) (Berman et al., 2003).

It is timely to reconsider the set of validation tools implemented by the wwPDB sites. As well as there being an order-of-magnitude more reference data than when most of the current tools were developed, this enriched database has informed our understanding of the features expected in protein structures, leading to the development of a number of powerful new validation tools that can detect a wider spectrum of problems and aid in their correction. At the same time, the recent decision by the wwPDB to mandate the deposition of underlying experimental data (structure factors for crystal structures, and restraints and chemical shifts for nuclear magnetic resonance [NMR]) creates new opportunities to develop rigorous tests of structure model quality. Despite widespread use of the conventional validation tools, there are still isolated instances of high-profile structures that are entirely incorrect (Chang et al., 2006), incorrect in essential features (Hanson and Stevens, 2009), or likely fabricated (Janssen et al., 2007; see also the highly commendable investigation by the University of Alabama at http://main.uab.edu/Sites/reporter/articles/71570/). Such instances, and the time it takes to uncover them, may reduce the confidence of the general user community in the quality of the PDB resource as a whole.

This paper reports conclusions drawn by the X-ray Validation Task Force (VTF) of the Worldwide PDB. These conclusions were reached through a workshop on “Next Generation Validation Tools for the PDB,” held at the European Bioinformatics Institute in Hinxton, UK from April 14–16, 2008, and through follow-up discussions. The goal of the workshop was to update the validation criteria that are used both by depositors when submitting new X-ray crystal structures to the PDB and also by users downloading structural data from one of the wwPDB sites. These criteria are also relevant to neutron, joint neutron/X-ray, and electron diffraction structures. The purely structural criteria should also be applicable to NMR and cryo-electron microscopy (cryo-EM) reconstruction structures, though the differing sources of error may change the relative importance of different validation tests. However, the experimental-data–based criteria are specific to the evaluation of single-crystal structures and are generally not applicable for evaluation of powder diffraction, cryo-EM reconstruction, NMR, or other structures not based on diffraction data.

The most obvious need for validation is to detect gross errors such as tracing the chain backward or building into a mirror-image electron density map. Such errors produce extreme outlier scores on most of the validation criteria presented below, and their cause could often be determined by a panel of technical crystallographic tests at deposition; if they could not be fixed, the authors presumably would choose not to deposit the structure. Less serious issues related to crystallographic data or refinement could prompt improvements by the depositor. Identifying the more local but serious errors in fitting side chains or backbone would contribute to further raising the overall accuracy of entries if they could be corrected before final deposition. Failing that, users should be alerted to possible problems. More generally, resolution-relative validation helps the depositor to judge how well the model approaches the best that could be achieved with the experimental data using current refinement methods and to catch slip-ups. Full-PDB measures help users to choose wisely among similar deposited structures, and local scores help them judge how much confidence they should place in specific features of interest to them. The high-profile cases of incorrect structures, discussed above, would all be flagged by the validation criteria recommended below.

As a novel measure to ensure the quality of published structures, we propose a new mechanism to make validation information available before publication. We propose that, at the time the PDB entry code is assigned, the depositor be given a summary validation report that can be made available to editors and referees. This report (probably in the form of a PDF) would include a brief summary of global quality indicators, as well as more detailed information that would allow one to judge whether specific conclusions are justified by the quality of the data and the model. Editorial boards of all journals that publish structural papers are encouraged to consider mandating the submission of a concise validation summary of well-established criteria to be shared with reviewers.

## Results and Discussion

Now that there are more than 70,000 entries in the PDB, statistical analysis can extract a tremendous amount of information about not only the mean values expected for various quantities, but also how they vary within a structure or across structures determined at different resolution limits.

Users of the PDB should be able to use the validation information for each deposited structure without a sophisticated understanding of all the validation tests that can be applied. The VTF recommends that the users' needs be served by presenting each validation criterion as a point on a distribution, in addition to reporting specific numeric scores. For scores that are well understood on the theoretical level, such as bond lengths and bond angles, the underlying distribution can be the probability of observing the values seen in the structure. However, many of the scores (such as the Ramachandran score) are obtained by a combination of theoretical insight and database mining. Such scores can be calibrated by the distribution of values seen over the whole PDB. Scores relative to distributions can then be presented as percentiles (which percentage of structures in the PDB are worse) or, after filtering to include only the most reliable structures, as RMS-Z values (see Experimental Procedures for details). Both percentiles and RMS-Z scores have the advantage that they place different criteria on a common scale and can be understood without having to remember target values for all of the individual criteria. On the other hand, as validation tools become even more widely adopted and refinement practice improves, the average quality of structures in the PDB will increase. This will raise the bar for new entries, but could also have the disconcerting effect that percentile scores for older structures drop over time.

As discussed in the section on presentation of results, some validation issues cannot be represented as a numerical score but are either present or absent in an entry. We recommend that these be presented as “concerns” or “unusual features.”

Below we discuss several types of validation criteria, including bonding geometry, conformation, molecular packing, fit to experimental data, and the quality of the data set itself. Distributions across the PDB are shown for validation criteria that are recommended for inclusion in the PDB validation report for referees and editors or in the validation analysis available to PDB users.

Note that when a validation criterion computed using a particular algorithm implemented in a particular program is recommended, other software implementing the same algorithm would be equally suitable after thorough testing.

### Geometric and Conformational Validation Criteria

Geometric criteria include bond lengths, bond angles, planarities, and chiralities; conformational criteria evaluate favorable combinations of backbone or side-chain torsion angles. Currently these are represented as rmsd values comparing the observed values to expectation for geometry, and as frequency of outliers for both geometry and conformation.

When the first important steps toward structure validation were taken in the early 1990s (Richards, 1988; Brändén and Jones, 1990; Jones et al., 1991; Laskowski et al., 1993), there were only about 1000 structures available in the PDB. With the subsequent massive expansion in the size of the PDB and improvements in our theoretical understanding, we now have a much better idea of what to expect in macromolecular structures. It is essential to use validation tools that update and extend their criteria in light of our improved knowledge.

Many of the potential geometric validation criteria are subjected to restraints or constraints by the refinement programs, so to some extent errors will be masked. Nonetheless, errors in fitting lead to strain that can be detected by residual errors in local geometric parameters such as bond lengths, bond angles, and planarity of groups of atoms. It should be noted that refinements at low resolution (poorer than 4Å) may not use all-atom refinement but rather restrict the refinement to rigid bodies or torsion angles only. Thus, geometric validation criteria based on bond lengths and bond angles may not be applicable to structures from some low-resolution refinements. Even at 3Å resolution, geometry restraints are often set more tightly, whereas at very high resolution they are sometimes turned off altogether. In all cases, however, an extreme local outlier indicates a local error of some sort. Combinations of torsion angles, such as the main-chain ϕ,ψ (Ramachandran) or side-chain χs (rotamers), are rarely restrained, so they remain extremely valuable for validation tests (Kleywegt and Jones, 1996).

#### Bond Lengths, Angles and Planes

Target values for means and standard deviations of bond lengths, angles, and planes can be obtained by analyzing the high-resolution, small-molecule structures in the Cambridge Structural Database (Allen, 2002). Nearly all refinement and validation software for proteins uses the values from Engh and Huber (1991). More recent compilations (e.g., Engh and Huber, 2001) have uncovered a few small modifications that could profitably be included in refinement but that affect the validation process very little, because bond length and angle deviations are considered serious outliers only when they are at least four or five standard deviations from their expected values. As the database of atomic resolution protein structures continues to expand, it is becoming possible to derive similar statistical data from protein rather than small-molecule structures. Validation based on such updated compilations would probably be preferable once they are available.

Global RMS-Z scores for bond lengths, bond angles, and planarity can help detect nonoptimal refinement procedures. For instance, an RMS-Z of 2.0 for bond lengths means that the bond length deviations from ideal target values are twice as large as those observed in the set of small-molecule structures from which the ideal values were derived. This indicates that the structure model would very likely benefit from refinement with an optimized weighting of the X-ray terms relative to the geometric restraints. In practice, the RMS-Z scores for geometry terms in well-refined structures at moderate resolutions are typically less than one because there is insufficient information in the diffraction data to compel the presence of large deviations (Joosten et al., 2009).

Individual bond-length outliers should be inspected, because at very high resolution they may reflect actual strained geometry that is functionally relevant. Otherwise, they usually indicate procedural rather than fitting errors and have only local impact. Small deviations in bond lengths that are consistent in direction, however, are quite important for detecting errors in unit cell dimensions; these are diagnosed in WHAT_CHECK (Hooft et al., 1996a).

**Figure 1:**
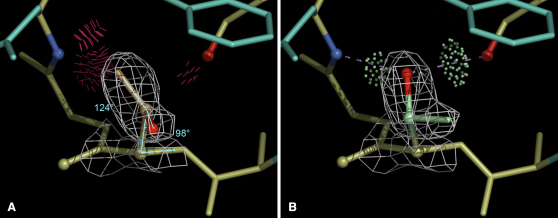
Correction of a Local Error for Thr 32 in PDB 1sbp, a Quite Good Older Structure at 1.7Å Resolution (A) This side-chain in 1sbp (He and Quiocho, 1993) has many serious all-atom steric clashes (clusters of red spikes) and no hydrogen bonds, and the tetrahedral angles at N-Cα-Cβ and at Cγ2-Cβ-Oγ1 (labeled) are bad outliers. (B) The side-chain has been turned 180° and now has ideal geometry, no clashes, two good hydrogen bonds, and a slightly better fit to the density.

In contrast, individual outliers in bond angles are of real interest for the interpretation of biology and structure, because they are frequently a symptom of serious local mis-fitting. An example is the backward-fit Thr in Figure 1A, which has atoms displaced by several Ångström and altered hydrogen bonding relative to the correct version shown in Figure 1B. This error can be diagnosed by two bond-angle outliers at 5σ and 7σ, as well as by steric clashes, a poor rotamer, and a large deviation of the Cβ atom from its ideal position relative to the backbone (Lovell et al., 2003).

All refinement programs to date assume that bond lengths and angles have a unimodal distribution, typically Gaussian. There is evidence that this can sometimes be too simple. For instance, the angle τ (N-Cα-C) shows a bimodal distribution depending on the secondary structure (Berkholz et al., 2009), several bond angles at the ribose ring are bimodal between C3′-endo and C2′-endo ring puckers (Gelbin et al., 1996), and some side-chain rotamers require a widening of bond angles at Cα and Cβ (Lovell et al., 2000). As more high-resolution data sets become available, refinement protocols may change, and this should eventually be reflected in the validation procedures as well.

The VTF recommends that percentile rankings lower than 0.1% for bond lengths, bond angles, and planarities be flagged as a “concern.” Individual geometry outliers with an absolute Z-score >5 should be recorded in the per-residue validation files and reports. Individual instances of inverted chirality should be flagged as a concern, unless the residue is identified by the depositor as a D-amino acid or a nonstandard nucleic acid sugar.

#### Protein Backbone Conformation

**Figure 2:**
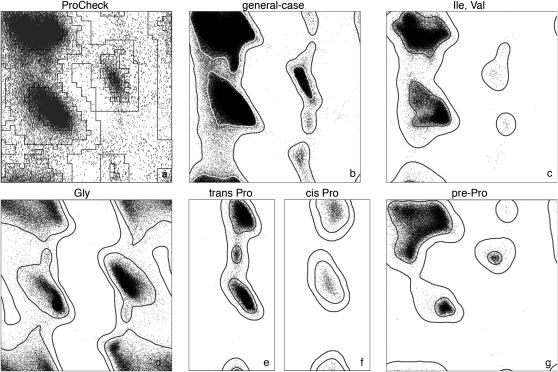
Ramachandran Distribution of ϕ,ψ Angles (A) The non-Gly, non-Pro distribution used in ProCheck, from about 100,000 residues of unfiltered data, plus the outlines for the ProCheck Favored, Allowed and Generously Allowed regions (taken from Morris et al., 1992). (B–G) The MolProbity-updated data distributions for the VTF-recommended 6 amino-acid categories, from about 825,000 residues after quality-filtering by resolution (<2Å), alternate conformations, and backbone B-factor (<30Å^2^). In (B–G), the inner contour encloses the favored 98% of the filtered data. The outer contour encloses 99.95% of the filtered data (all but 1 in 2000, or equivalent to 3.5σ), now feasible for individual categories as well as the general (Chen et al., 2010); this contour is taken to divide Ramachandran outliers from allowed conformations. See also Figure S1.

The program PROCHECK (Laskowski et al., 1993) was the first widely-used tool for the validation of protein structures and the first to introduce Ramachandran criteria (developed from the backbone treatment in Ramachandran et al., 1963), and it had an important impact on the quality of structures subsequently released. However, when PROCHECK was developed in 1993, it was possible to obtain 100,000 observations of non-Gly, non-Pro ϕ,ψ values only by including all residues of all entries in the contemporaneous PDB. The noise introduced by poor structures or high crystallographic B-factors made interpretation difficult, as can be appreciated from the plot reproduced in Figure 2A along with the familiar PROCHECK favored, allowed, and generously allowed regions. Over the following decade, it became feasible to filter by homology, resolution, and B-factor, and a number of improved Ramachandran measures were developed, such as for O (Kleywegt and Jones, 1996) and for WHAT_CHECK (Hooft et al., 1996a), which included a procedure for annual updates of secondary structure-specific Ramachandran distributions. These Ramachandran distributions had converged on the outline of the “favored” regions, now generally taken as including 98% of the high-quality data. When the database had grown to 100,000 quality-filtered residues, boundaries could also be defined for 3.5σ outliers, in the general case, as done in MolProbity (Lovell et al., 2003; Davis et al., 2004).

Since the mid-1990s, the database has grown by an order of magnitude, allowing even finer-grained local evaluations. Figure 2B shows a more restricted general-case (non Gly/Pro/Ile/Val, non pre-Pro) Ramachandran plot for 582,000 quality-filtered residues from 4400 nonhomologous PDB files at <2Å resolution, with contours from density-dependent smoothing. (Figure S1 [available online] presents the evidence for grouping the general-case residues.) Some regions previously considered as disallowed are now seen to be acceptable, and vice versa. The most notable change is the clear separation of relatively rare (presumably strained, but possible) conformations in the less favored “allowed” regions between the two contours from those in completely disallowed regions with almost no quality-filtered data points at all. More than half the plot area is empty after quality filtering, with only 1 in 2000 residues outside the outer contour. Figures 2C–2G show separate Ramachandran plots for residue categories with significantly different distributions: Ile/Val (Figure 2C), Gly (Figure 2D), trans-Pro (Figure 2E), cis-Pro (Figure 2F), and pre-Pro for residues that precede prolines (Figure 2G) (Lovell et al., 2003). Each residue can be assigned to only one class by using the order of precedence: Gly,Pro > pre-Pro > Ile/Val > general.

**Figure 3:**
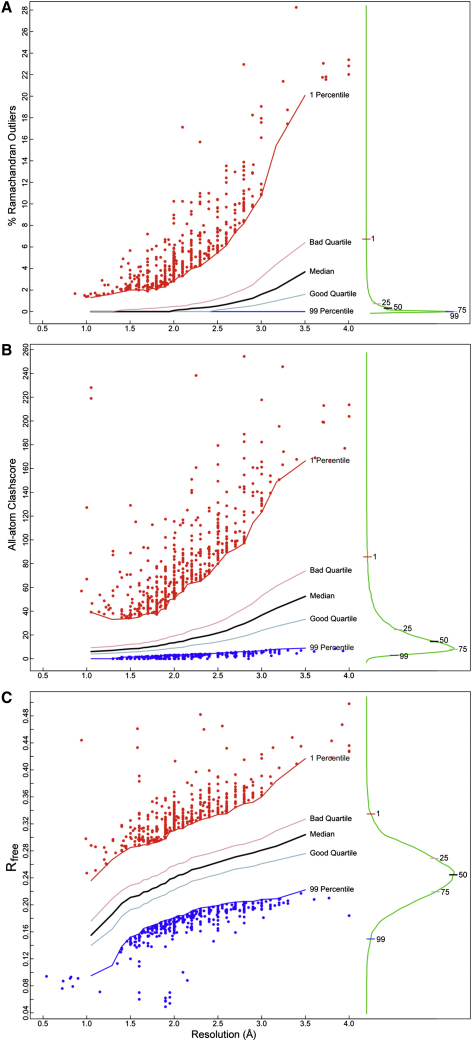
All PDB (X-ray, since 1990) Distribution of Validation Criteria as a Function of Resolution Median and quartile levels are plotted smoothly, along with all individual data points for outlier structures beyond the 1st percentile (poor; red) or the 99th percentile (good; blue) values (see Supplemental Information for detailed criteria, and for procedures and discussion of these shingle-smoothed, quartile-and-outer-percentile plots with outlier datapoints). At the right of each panel is the resolution-independent, 1-D distribution (green line) with median, quartile, and outer percentile values marked, for the aggregated set of all PDB entries. (A) Percent Ramachandran outliers. (B) MolProbity clashscores. (C) R_free_. See also Figures S2 and S3 and Table S1.

**Figure 4:**
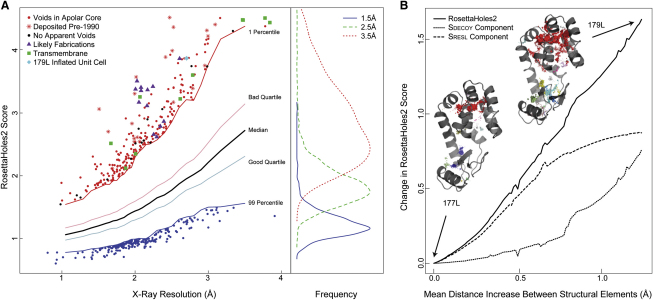
RosettaHoles2 Scores (A) RosettaHoles2 scores for crystal structures in the PDB as of May 14th 2010; only structures that contain primarily protein, have no missing nonhydrogen atoms, and are larger than 10 kDa are included. Structures marked in red contain at least one large void surrounded by hydrophobic side chains, 18 structures without such voids are marked with black circles, transmembrane proteins are marked with green squares, and the retracted structure 179L is marked with a cyan diamond. Structures marked as purple triangles have been identified as likely to arise from fabrication. On the right is a histogram of scores for 1.5Å, 2.5Å, and 3.5Å resolution bins. (B) A more detailed examination of the 179L cell parameter error, showing the voids present in 177L and 179L, which are identical except for the error. The degradation of the RosettaHoles2 score and its two components is shown for increasing void sizes.

Despite the clean plots shown here, Ramachandran outliers are quite common in unfiltered PDB entries. Although it is still possible that an individual ϕ,ψ outlier is correct, outliers should always be examined by the structural biologist, in the context of experimental data such as electron density, and should be treated with great caution by the end-user. The percentage of Ramachandran outliers is an excellent measure of structure accuracy that correlates strongly with resolution. Nonetheless, even at resolutions worse than 4Å, excellent Ramachandran statistics can be obtained using full-atom refinement if accurate structures are available for domains of the overall structure (e.g., Davies et al., 2008). Outliers (beyond the 99.95% contour) on all six distributions can be combined to give an overall percent Ramachandran outliers for a given structure. The distribution of percent Ramachandran outliers across the entire PDB (X-ray, since 1990) is shown both globally and as a function of resolution in Figure 3A, with smoothed lines for median, quartile, and extreme percentiles. Relative rank (percentile) for a structure's score within its resolution range is a good measure for comparative quality (Figure S2 and Table S1 provide details of how the smoothing was performed; Figures 3, 4A, and S3 also present similar distributions for other validation criteria discussed below).

The VTF recommends that the summary validation for each PDB entry containing a protein include the residue category–specific Ramachandran outlier frequency at the level of 1:2000 (∼3.5σ), available from MolProbity, expressed both as a percent of total residues and as percentile ranks globally and within the resolution class. Individual outliers identified at the same level should be flagged in the per-residue validation file. Presenting the entry's six Ramachandran plots should be considered if feasible, either on a linked web page or for the referee report.

#### Protein Side-Chain Conformation

Similar considerations and methodologies apply to the multidimensional distributions of χ side-chain torsion angles, whose favorable combinations define side-chain rotamers, a concept first introduced by Ponder and Richards (1987). A large number of rotamer libraries (lists of the discrete, minimum-energy conformations) have been developed for protein design and prediction, often with an additional grid of sample points; one of the most widely used is from Dunbrack and Cohen (1997). For validation purposes, PROCHECK uses binned χ1χ2 plots, O calculates the root-mean-square deviation (rmsd) to the most similar rotamer (Jones et al., 1991), WHAT_CHECK uses continuum statistics for χ1χ2 (De Filippis et al., 1994), and MolProbity uses smoothed distributions in all χ dimensions, increasing sensitivity to the strong multidimensional couplings. Like most other validation criteria, rotamer quality varies with resolution and especially with B-factor. A large fraction of surface side-chains assume multiple conformations, but each of those conformations is expected to be rotameric because there are no rigid interactions to hold the side chains in an unfavorable conformation. Indeed, a systematic variation with resolution of mean χ1 angles can be explained by the existence of unmodeled multiple rotamers (MacArthur and Thornton, 1999).

The available data are more limited for individual rotamers than for the grouped Ramachandran torsions, because each amino acid is a separate case and may have as many as four χ angles. Therefore, we can currently define disfavored or poor rotamers that are seen infrequently (<1%) with low B-factors in high-resolution structures, but we cannot yet reliably distinguish those from true outliers (taken to require at least a 3σ Z-score). For instance, an eclipsed χ angle is not rotameric, but is on occasion genuinely stabilized by multiple H-bonds. An additional complication is that rotamer distributions can be distorted by systematic errors caused by fitting the end of a side chain 180° backward into its local electron density (as happened to the Thr in Figure 1A); these are not entirely removed by applying d_min_ and B-factor filters to the reference data (Lovell et al., 2000; Headd et al., 2009). Nonetheless, the percent of infrequent rotamers is a sensitive test for the overall quality of a structure model. The distribution of poor rotamer frequency across the entire PDB is plotted in Figure S3A, both globally and as a function of resolution.

The rotamer evaluation implemented in MolProbity is perhaps the most suitable system currently available for validation purposes because it uses smoothed full distributions for all χ angles and deals with some of the systematic errors. The VTF recommends that this algorithm be adopted initially. As soon as is feasible, however, larger current datasets should be used to update side-chain χ distributions, eliminating “decoy” systematic errors either by explicit analysis or by more strict filtering, and working toward distinguishing between strained and impossible side-chain conformations.

The VTF recommends that the percent of infrequently observed (probability <1***%***) rotamers and the percentile rankings both globally and within the resolution class be reported in the validation summary, and that individual poor rotamers be flagged in the per-residue validation data.

### Validating Atomic and Molecular Interactions

The study of high-resolution, well-refined structures shows that the folding of macromolecules brings together surfaces that are highly complementary in shape, charge, and hydrophobic character. Because such complementarity is usually not a direct target of current refinement methods, validation tools based on assessing intra- and intermolecular interactions can be extremely powerful in detecting potential errors.

#### All-Atom Contacts

The most straightforward sort of contact problem in a structural model is a physically impossible overlap, or clash, of nonbonded atoms. Refinement strongly penalizes clashes between nonhydrogen atoms, so those occur only rarely. On the other hand, hydrogen atoms have not traditionally been modeled explicitly in macromolecular refinement (though there is evidence that including them is beneficial [Sheldrick & Schneider, 1997], and there is a recent trend to including them), because they are not visible in the electron density except at very high resolution. However, the hydrogen atoms are of course present in the actual molecule and are only slightly “softer” than the heavier atoms, so that the requirement for their favorable contacts provides a sensitive and powerful validation tool (Word et al., 1999a; Chen et al., 2010).

Most H atoms can be placed by simple geometry with adequate accuracy, but the orientations of groups such as OH, NH_3_, and even side-chain amides need to be optimized within entire (local) H-bond networks, including their interactions with nonpolar as well as polar atoms and with ordered waters (Hooft et al., 1996b; Word et al., 1999b). That optimization process has the beneficial side effect of diagnosing incorrect 180° flips of Asn, Gln, and His (NQH) side-chains, and of robustly correcting them from H-bond and all-atom contact evidence without affecting agreement to the diffraction data (Word et al., 1999b; Higman et al., 2004; Arendall et al., 2005). The NQH flip corrections provide a no-cost route to local but often important improvements to model accuracy, especially at resolutions better than ∼3Å. Reporting an NQH flip score would encourage the use of that route. Note that methyl groups are not rotated in the H atom optimization. Although other contacts will sometimes cause methyl groups to deviate slightly from a staggered conformation, the false-positive rate (in low-B regions of very-high-resolution structures) is extremely small for clashes ≥ 0.4Å. Nearly all serious methyl clashes result from misplaced C atoms, most often because of backward-fit side-chains, as in Figure 1 (Word et al., 1999b; Arendall et al., 2005; Headd et al., 2009).

Once hydrogens are present, the all-atom contacts can be calculated (Word et al., 1999a) for H-bond, favorable van der Waals, and clash components. For validation purposes, their most telling and widely adopted uses have been (1) lists or visualizations of individual bad clashes (defined in MolProbity as unfavorable atomic overlaps ≥0.4Å) and (2) the overall “clashscore,” which is the number of bad all-atom clashes per thousand atoms. The all-PDB distribution of clashscore versus resolution is shown in Figure 3B, demonstrating the high correlation of an all-atom clashscore with resolution. Of the 20 lowest-percentile outlier structures after filtering by date, seven involve waters modeled impossibly close to protein or nucleic acid atoms (such as 1rb1, now replaced by 3k7z), three result from tests of novel methods, three are early nucleic acid structures with poor backbone conformations, two are membrane proteins, and five are irregular models at lower resolutions with outliers on many criteria. Retracted structures (not plotted here) nearly all score in the poorest percentile. In all of these cases, the validation measure flags something that is indeed seriously wrong with the model.

Individual all-atom clashes are even more important than the global clashscore for both producers and users of crystal structures, because they are directional as well as local (see Figure 1) and are thus very helpful for identifying (and rebuilding) problem areas in proteins, nucleic acids, and interfaces. At resolutions up to about 2.5Å, it is possible to correct 70%–90% of clash, geometry, rotamer, and Ramachandran outliers, producing modest improvements in R and R_free_ (Arendall et al., 2005). Individual outliers should therefore be reported to enable both corrections by the depositor and evaluation of local reliability by the end-user.

#### Underpacking

Side-chain packing in the core of real protein molecules is exquisite. Steric clashes provide a sensitive measure of local overpacking (see above), but assessing underpacking has been more difficult. The quality of protein core underpacking can now be assessed visually and quantitatively using RosettaHoles2, a refinement of RosettaHoles (Sheffler and Baker, 2009), particularly suited to structure validation (Sheffler and Baker, 2010). The RosettaHoles method fills voids by placing the largest nonintersecting sphere next to each atom, removes those accessible to solvent, and analyzes the distribution of surface area around each cavity at a range of probe sizes. RosettaHoles2 adds a differentiable score function trained to distinguish high-resolution crystal structures from predicted models and is found also to distinguish NMR and low-resolution structures from high-resolution.

Figure 4A shows the RosettaHoles2 packing score for crystal structures in the PDB. The packing score correlates roughly with resolution and is calibrated so that the RosettaHoles2 score should be less than the resolution (d_min_) in most cases. Structures in the lowest percentile were examined individually, and all but 18 contain voids significantly larger than a water molecule surrounded by hydrophobic residues. More than 6% of transmembrane proteins are in the lowest percentile as a result of water-filled polar channels in their cores and a preponderance of hydrophobic surface residues. Structures that are likely fabrications show up as clear outliers on the plot.

RosettaHoles2 cartoon representations of cavities in 177L and 179L (retracted) are illustrated on Figure 4B, showing that 179L has excessive void volume (red voids). The stretched structure 179L was caused by using incorrect cell constants with ∼10% error in the *a* and *b* cell parameters (Dale Tronrud, personal communication). Depending on how restraints are treated in refinement, the effects of a cell dimension problem can be manifested mainly in packing or in bond-length deviations that are systematic in the directions of cell edges; the former can now be detected with RosettaHoles2 and the latter are diagnosed by WHAT_CHECK. It is possible that some of the other packing outliers in Figure 4A are also caused by cell parameter errors (Tronrud and Matthews, 2009).

To illustrate the sensitivity of RosettaHoles2 to small and large packing defects, a set of intermediate structures was produced by interpolating between 177L and 179L, which are nearly identical aside from the unit cell dimensions used in refinement. The increase in RosettaHoles2 score is approximately linear with the increase in separation between structural elements (Figure 4B). Also plotted are the two components that make up RosettaHoles2, a regression based S_RESL_ score that detects small flaws correlated with X-ray resolution, and a discrimination-based S_DECOY_ score that detects major flaws not typically observed in crystal structures of any resolution.

#### Hydrogen Bond Quality

Hydrogen bonds are key interactions for specifying protein secondary and tertiary structure. Unsatisfied, buried hydrogen bond donors and acceptors mark a loss of hydrogen bonding potential and are uncommon in high-quality structures (Hooft et al., 1996b). A measure of buried, unsatisfied hydrogen bond donors/acceptors, implemented in WHAT_CHECK, provides a test complementary to over- and underpacking, because it measures the plausibility of specific packing interactions. In WHAT_CHECK, hydrogen bond donors or acceptors are judged to be buried if they have an accessible surface area <0.5Å^2^. They are judged to be unsatisfied if they are not involved in a hydrogen-bonding interaction with a calculated energy >10^−8^ kcal/mole; crudely, this means that they have no complementary partner within a cutoff distance of 3Å. Figure S3B shows the distribution of the fraction of buried hydrogen bond donors/acceptors that are unsatisfied, both globally and as a function of resolution.

The VTF recommends graphical display in the validation summary of the resolution-relative and all-PDB percentile rankings, plus listing of absolute numerical scores, for the global measures of all-atom clashscore (for all structure types), RosettaHoles2 (or similar) packing score, and fraction unsatisfied buried H-bonds (for proteins). Individual all-atom clashes and individual unsatisfied H-bonding groups should be reported in the per-residue validation data.

### Structure-Factor and Electron-Density Validation

When only atomic coordinates were available for most PDB entries, it was possible to detect the existence of some problems with the underlying diffraction data, but almost impossible to pinpoint them precisely, let alone fix them. Now that structure-factor deposition is mandatory for new entries, much richer information is available to the user. The availability of structure factors enables the use of tools for assessing the global quality of crystal structures and, probably of greater importance, the local quality-of-fit to the electron density.

In addition, the availability of structure factors allows data quality analysis in which the presence of experimental problems or artifacts can be assessed. These problems can be flagged for users as potential “concerns” or “unusual features” including the likely presence of twinning, translational noncrystallographic symmetry (NCS), anisotropic diffraction, data incompleteness, and potential outliers. Convenient collections of structure-factor–based tests have been implemented in SFCHECK (Vaguine et al., 1999) and phenix.xtriage (Zwart et al., 2005a).

X-ray diffraction data typically conform to expected distributions of intensities (Wilson, 1949). In certain cases, some of them pathological, these intensity distributions are perturbed; for example, merohedral twinning leads to changed intensity distributions and must be accounted for appropriately in structure solution (see Parsons, 2003 for a review of twinning phenomena). Multiple tests can be performed before the availability of an atomic model, thus permitting a fundamental validation of the experimental data, and further tests exploit information from the atomic model. A comprehensive set of tests has been implemented in the phenix.xtriage program (Zwart et al., 2005a), which is part of the Phenix software (Adams et al., 2010). A subset of these tests is also available in other programs, such as the CCP4 (CCP4, 1994) programs Truncate (French and Wilson, 1978) and SFCHECK (Vaguine et al., 1999). These tests can be important to understanding features of the atomic model and its fit to the experimental data. The tests relevant to validation of protein structure depositions, with emphasis on those tests that are most useful subsequent to structure determination, are discussed below.

#### Wilson Plots, Outliers, and Translational NCS

**Figure 5:**
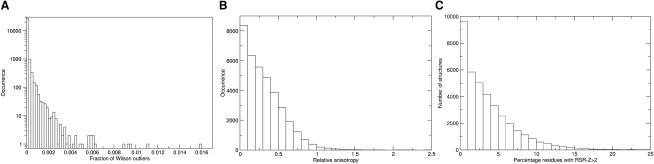
Histograms Showing Distributions of Validation Criteria Computed with X-Ray Diffraction Data (A) Histogram showing the numbers of structures with different fractions of Wilson outliers. Datasets showing evidence of translational NCS (nonorigin Patterson peak >20% of the origin peak) have been omitted. Note the logarithmic scale on the vertical axis. (B) Histogram showing the numbers of structures for which the data show different levels of relative anisotropy. (C) Histogram showing the numbers of structures with different percentages of residues having RSR-Z > 2 (i.e., much poorer than average fit to density). The good quartile boundary is 1.0, the median is 2.7, the poor quartile boundary is 5.3, and the 1st percentile is 16.3. Approximately 11% of structures have no residues with RSR-Z > 2.

The conventional Wilson plot, which shows the logarithm of the normalized mean intensity as a function of resolution, is remarkably consistent in shape for a wide variety of protein diffraction datasets (Popov and Bourenkov, 2003); a different curve should be used for nucleic acid datasets (Zwart et al., 2005a). Deviations from the expected curve, such as too high a mean intensity at low resolution, or increasing mean intensity at high resolution, can indicate problems with data processing. In xtriage the data are compared with an empirical Wilson plot that has been derived using more than 2500 high-resolution datasets obtained from electron density server (EDS) (Kleywegt et al., 2004). Individual potential outliers in the experimental data can be identified by analysis of normalized intensities (Read, 1999). The xtriage program assigns probabilities to the largest normalized intensities using basic extreme value statistics (Dudewicz and Mishra, 1988) and reports very unlikely observations. The presence of outliers does not invalidate the entire set of experimental data; for example, diffraction measurements close to the beam stop may be systematically perturbed. In addition, the intensity distribution is perturbed in the presence of translational NCS. However, the presence of many outliers may indicate a fundamental problem with the data. Figure 5A shows the distribution of the percentage of reflections flagged as outliers in the PDB. The VTF recommends that the presence of more than 0.1% (1 in 1000) outliers should be flagged as a “concern” for datasets not deemed to be affected by translational NCS; fewer than 1% of datasets would be flagged at this threshold. Further work will be required to develop tests for outliers in the presence of translational NCS.

A likelihood-based method can be used to estimate the overall anisotropic Wilson B tensor (Popov and Bourenkov, 2003), even when only low-resolution data are available (Zwart et al., 2005a, 2005b). An analysis of experimental datasets deposited in the PDB shows that 13% of deposited X-ray datasets have an anisotropic ratio ([B_max_-B_min_]/B_mean_, where B_min_, B_max_, and B_mean_ are computed from the B-factors associated with the principal axes of the anisotropic thermal ellipsoid) >0.5, and only 1% have an anisotropic ratio >1 (Figure 5B). Correction of the data for anisotropy, which perturbs the intensity distribution, is important for the subsequent calculation of intensity-based statistics to detect features such as twinning (Yeates, 1997). The VTF recommends that an anisotropic ratio >1 be flagged as an “unusual feature.”

Given an atomic model, it is possible to determine whether diffraction data are in the form of intensities (I) or amplitudes (F) based on R-factors between the model and the dataset. This check of data type should be performed at the time of structure deposition to ensure that the data labels are correct. The analysis can be performed using the model_vs_data program (Afonine et al., 2010) within the Phenix software. The VTF recommends that, when it appears that the deposited diffraction data have been mislabeled but the labeling is not corrected by the depositor, this should be noted as a “concern” in the validation report.

It is common for more than one copy of a macromolecular entity to be present in the crystallographic asymmetric unit. In some cases, these molecules may be principally related by translation. Such a translation can have a profound impact on the measured diffraction intensities, leading to systematic modulations in reciprocal space (Kleywegt and Read, 1997). These modulations can make structure solution and refinement challenging, because they lead to a breakdown of some of the underlying statistical assumptions of modern maximum likelihood methods. Translational NCS can be detected by the presence of large nonorigin peaks in a native Patterson map. Analysis of the PDB indicates that 8% of structures show a peak in the native Patterson map ≥20% of the origin. The probability of a macromolecular dataset showing such a tNCS peak height and not possessing tNCS is <1% (Zwart et al., 2005b). We can define very strong tNCS as a probability of observation <10^−6^ (i.e., one in a million), which corresponds to a peak height of 75%. The presence of very strong tNCS can indicate a misassignment of crystallographic symmetry, where a centering operation has been missed during data processing (Sauter and Zwart, 2009). If this is detected by phenix.xtriage, the translation operator is combined with the input crystal symmetry and any possible higher symmetry is reported. The VTF recommends that the presence of a very strong peak in the native Patterson function (≥75% of the origin peak) be flagged as a “concern” if the position and height of the peak are consistent with an unidentified centering operation, or as an “unusual feature” if it is not and has been identified by the depositor as an NCS operation rather than a crystallographic centering operation that results in improved agreement with the diffraction data. By this criterion, 0.6% of entries in the current PDB potentially have an unidentified centering operation.

#### Missed Symmetries

During the processing of diffraction data it is possible for one or more crystallographic symmetry operators to be missed, *i.e.* the assumed point-group symmetry is lower than the true point-group symmetry. If undetected it is possible for structural differences between molecules to be misinterpreted (Kleywegt et al., 1996). However, there are also many examples where refinement in a lower symmetry setting produces significantly improved agreement with the diffraction data in terms of R_free_ and other criteria. Care must be taken that the set of Miller indices used for cross validation (R_free_) is compatible with the symmetry of the lattice. If not, real or pseudo-crystallographic symmetry (and twinning) will introduce statistical dependencies between the working and validation sets, artificially lowering the R_free_.

The crystallographic lattice parameters can be analyzed to determine whether higher symmetry is possible, then reflections related by potential symmetry operators can be compared to determine whether each operator is close to crystallographic. Such analyses are carried out by phenix.xtriage (Zwart et al., 2006), LABELIT (Sauter et al., 2006), and pointless (Evans, 2006). Perfect twinning also leads to apparent higher symmetry. Therefore, it is advisable to also check the atomic model for potential higher symmetry, either by examining the coordinates, as done in WHAT_CHECK (Hooft et al., 1996a) or LABELIT (Poon et al., 2010), or the calculated structure factors with the RvR test (Lebedev et al., 2006). If higher symmetry is found to be likely, rerefinement of the model against the appropriately merged data may be recommended. Analysis of the PDB suggests that higher symmetries may be possible for 2% of current entries (Poon et al., 2010). However, a very careful analysis can be required to distinguish between, for instance, missed symmetry and pseudosymmetry combined with twinning. The VTF recommends that the presence of potential higher symmetry be flagged as an “unusual feature.”

#### Twinning

Merohedral twinning in macromolecular crystallography can occur when the lattice supports a higher symmetry than the true underlying symmetry of the crystal (reviewed by Yeates [1997] and Parsons [2003]). For example, a P3 lattice can support the highest symmetry of P622, or a monoclinic lattice with the cell angle β equal to 90° supports higher orthorhombic symmetry. The presence within the crystal of multiple domains, related by rotational symmetries, leads to superposition of Bragg reflections in reciprocal space and summation of intensities. These summed intensities lead to problems with structure solution and refinement, typified by high R and R_free_ values. However, if twinning is detected, it is possible to perform structure refinement using a twinned target function that explicitly accounts for the twinning (Yeates, 1997).

A number of tests have been developed to detect possible twinning from the distributions of intensities (Yeates, 1997; Padilla and Yeates, 2003). The availability of an atomic model at deposition time makes it possible to calculate model-based twinning statistics. These can provide other insights into the experimental data, for example the RvR test (Lebedev et al., 2006). If twinning is detected but has not been taken into account in structure refinement, then it is appropriate to repeat the final cycles of refinement, especially the interpretation of solvent peaks, using a twinned target.

The VTF recommends that the probable presence of twinning be flagged as an “unusual feature,” *i.e.*, when an estimated twin fraction of >5% is calculated from the data. This condition is met by 2.5% of entries in the current PDB. The VTF further recommends that if the estimated twin fraction is >20% and there is no indication that twinning was accounted for in the refinement target, this should be flagged as a “concern.” As is the case for missed symmetries, it is of vital importance that the free set of Miller indices be invariant under the twin law.

#### Agreement of the model with the diffraction data

The current criteria for fit of crystal structures to diffraction data are the conventional crystallographic R factor, R_free_ for a control subset of data (Brünger, 1992), and the real-space residual, which quantifies the fit of the model to electron density (Jones et al., 1991). R_free_ is generally considered the most useful global measure of model-to-data agreement. Figure 3C shows the distribution of R_free_ for all PDB entries, with R_free_ defined as a function of resolution and over the entire PDB.

A useful way to relate the local details of an atomic model to the experimental data is the use of real-space fit statistics, often assessed as per-residue plots. Jones introduced the real-space R-value (RSR) in the early 1990s (Jones et al., 1991) as a quantitative measure of the fit of model and density. The RSR value of a residue, ligand, or other entity is calculated by first defining an envelope of points in the vicinity of the entity's atoms. The “observed” density (typically, a σ_A_-weighted (2mF_o_-DF_c_, α_c_) map; Read, 1986) is then compared, point-by-point, with a calculated density. In the original implementation (Jones et al., 1991), the calculated density was evaluated as a sum of Gaussians, one for every atom. In the EDS server (Kleywegt et al., 2004), a σ_A_-weighted (DF_c_, α_c_) map is used instead. RSR is then calculated as $\sum \left|\rho_{obs}-\rho_{calc}\right|/\sum \left|\rho_{obs}+\rho_{calc}\right|$, where the sums extend over all grid points covered by the envelope. One disadvantage of the RSR calculation is that the two maps must be scaled together; this can be circumvented by calculating a real-space density correlation coefficient (RSCC) instead, using the same set of grid points. The RSCC in turn has the disadvantage of being insensitive to the density levels; for instance, a very weak, but spherical density would correlate well with a calculated map if a water molecule was positioned there in the model. Other real-space fit measures have been proposed as well (Vaguine et al., 1999). The VTF recommends that measures based on RSR should currently be chosen over alternatives such as RSCC for validation, primarily because there is more experience with RSR. Figure 5C shows a histogram of the percentage of residues with RSR-Z > 2 for the entire PDB. Because RSR-Z is normalized for the resolution shell, this statistic does not vary with resolution and thus can only be used to judge the relative quality of a PDB entry, but not its absolute quality.

A plot of the RSR value as a function of residue number provides a quick impression of the areas that fit the density relatively well or relatively less well, and indeed such plots are available for tens of thousands of macromolecular crystal structures from EDS. However, as with RSR values, the average RSR values for good models tend to be considerably smaller at higher resolution. In addition, certain types of residues can be expected to have systematically higher or lower RSR values than others (*e.g.*, glutamates are often found on the surface of proteins and thus have comparatively poor electron density). For this reason, EDS from its inception has gathered RSR statistics for all common amino acid and nucleotide types, in a number of ranges of resolution. Using the tabulation, for the relevant resolution bin, of the mean RSR for each residue type and the standard deviation from the mean, an RSR Z-score can be calculated for every residue in a given protein or nucleic acid (see Supplemental Experimental Procedures for the definition of a Z-score). If a large fraction of residues has positive RSR Z-scores, it means that the model, on average, does not explain the experimental data as well as one would expect at the given resolution. In EDS, for every chain, the percentage of residues that have RSR-Z > 2 is reported. For example, for the now obsolete entry 1F83, the percentage of residues with RSR-Z > 2 is 10% for the enzyme model, but 96% and 100% for the two parts of the peptide model, highlighting the lack of experimental evidence for peptide binding that led to the retraction of this structure (Hanson and Stevens, 2009).

The VTF recommends that the validation summary for each PDB entry display the global and resolution-specific percentile ranking of R_free_ and list the absolute value of R, R_free_ and the percentage of residues with RSR-Z > 2. The individual-residue RSR-Z scores should be reported in the per-residue validation file.

### Validating Nonprotein Components

Because proteins comprise the great majority of the content of the PDB, it has taken longer to accumulate enough information on the nonprotein components to develop statistical tools to validate their structure.

#### Nucleic Acids

DNA and RNA structures, either by themselves or complexed with protein, are of course subject to the same tests of data and of real-space residuals described above, with a few caveats such as differences in the expected shape of the Wilson plot (Zwart et al., 2005a). Bond lengths and bond angles are slightly affected by sugar pucker, but 4σ outliers can be suitably evaluated from standard values (Parkinson et al., 1996), and occasional incorrect chirality of sugar substituents or strong deviation from base planarity can be flagged, with proper attention to modified bases such as dihydrouracil. One interesting difference from proteins is that the electron density effectively has more contrast in nucleic acids, with the dense symmetric phosphates and the large planar bases giving rise to very clear density features, whereas the rest of the backbone and the sugar pucker are much less distinct and have many variable torsion angles that are difficult to determine correctly at the 2.5–3.5Å resolution typical of large RNAs or complexes. Fortunately, the addition of hydrogens and calculation of all-atom contacts is very sensitive to problems along the backbone, making the all-atom clashscore (discussed above) an important validation measure for nucleic acids. Both RNA and DNA bind many and varied metal ions, at full or partial occupancy, which are not easy to distinguish from one another or from waters. When well-accepted validation tests for ions become available, they should be adopted by the wwPDB.

The specific conformation of the sugar-phosphate backbone is much more variable in RNA than in DNA, and is central in many biological functions of RNA such as catalysis, aptamer recognition, and drug and protein binding. Therefore, some further definitions and tests of ribose pucker and of backbone torsion-angle conformations have been collaboratively developed by the RNA Ontology Consortium (Richardson et al., 2008). In RNA, the ribose ring pucker is nearly always very close either to C3′-endo (as in A-form helices) or to C2′-endo. These two puckers can be distinguished by the geometrical relationship between the base plane and the following (3′) phosphate, but in deposited structures the less common C2′-endo puckers are fairly often incorrectly fit as C3′-endo, whereas other less favorable pucker states also occur. The percent of unlikely ribose puckers correlates well with resolution and is close to zero in small-molecule structures and well under 1% for high-resolution RNA entries.

The six torsion angles along the RNA backbone adopt distinct conformers (analogous to protein side-chain rotamers), especially when analyzed within the “suite” grouping from sugar to sugar rather than within the chemical residue from phosphate to phosphate. The multidimensional distributions of favorable RNA backbone suite conformers are known (54 of them currently recognized), and the percent of unfavorable suite conformers is a useful validation measure (Richardson et al., 2008). These criteria are already very helpful, but will improve in precision and robustness as the database of nucleic-acid structures continues to grow.

The VTF recommends that the data, real-space, geometry, and clashscore metrics are treated essentially the same for nucleic acid and complex structures as for proteins. The RosettaHoles2 packing score is not suitable, and side-chain rotamer and Ramachandran criteria are not applicable to nucleic acids. For RNA-containing structures, the percent of unlikely ribose puckers and of unfavorable backbone suite conformers should be listed numerically, the global and resolution-specific percentile ranks displayed for percent unlikely ribose puckers, and the individual scores flagged on per-residue plots.

#### Carbohydrates

About 7% of PDB entries contain carbohydrate residues, covalently bound in glycoproteins or noncovalently bound in protein-carbohydrate complexes (Lütteke, 2009). Unfortunately, there is a rather high rate of error within the carbohydrate moieties of PDB entries (Crispin et al., 2007; Lütteke and von der Lieth, 2004; Lütteke, 2009; Nakahara et al., 2008), because depositors frequently have poor knowledge of carbohydrate structure. The names of carbohydrates depend on chirality, which is subject to coordinate errors if refinement restraints are inappropriate. As a result, many errors arise from mismatches between the PDB residue names and the residues actually present in the 3D structures. Carbohydrate residues were renamed during the PDB remediation process (Henrick et al., 2008) so that the residue names match the coordinates in the remediated entries. However, mismatches between residue names and coordinates can be found again in entries that have been released after the remediation date. Moreover, the mismatches are not always based on the selection of wrong residue names but can also be caused by errors in the atomic coordinates. In the latter case, the errors are masked by the renaming of residues in the remediated entries: if a residue name is changed to match erroneous coordinates, the inconsistencies are difficult to detect.

Biological pathway information can be used to identify erroneous coordinates for N-glycans (Nakahara et al., 2008), and to some extent also for O-glycans. For noncovalently bound ligands (regardless of whether they are carbohydrates or any other ligand), however, often no comparable information is directly available. The PDB Carbohydrate Residue check (pdb-care) tool (Lütteke and von der Lieth, 2004) was developed to aid researchers in the validation of carbohydrate residues in PDB entries. The original version searches mainly for inconsistent residue notation and for unusual bond lengths; a recent update also implements a validation of N-glycan chains to detect residues that are not known to occur naturally.

The geometry of glycosidic linkages can be analyzed using ϕ,ψ-plots similar to the Ramachandran plot. Such plots can be created by the Carbohydrate Ramachandran Plot (CARP) software (Lütteke et al., 2005). Outliers in the CARP plots are not necessarily erroneous, because interactions of the carbohydrate chain with the protein part of the PDB entry might induce a conformation other than the one preferred by the uncomplexed carbohydrate. Nevertheless, the plots can help researchers locate potential problems within the carbohydrate moieties.

The VTF recommends that outliers in carbohydrate nomenclature and “unusual” residues within the N-glycan core region be flagged as “concerns.”

#### Ligands

In recent years, there has been a marked shift in the way protein structures are studied. Where earlier the structure of the macromolecule itself was the main object of investigation, it is now commonplace to study the structures of large numbers of complexes with a variety of small-molecule ligands, including co-factors, inhibitors, substrate analogs, products, crystallization additives, etc. This presents a number of problems: to obtain starting coordinates for the ligand; to obtain an appropriate refinement dictionary for it, including proper bond-distance and angle restraints; and to find methods to validate it. Solutions to the first two problems have been suggested (Kleywegt, 2007; Kleywegt et al., 2003), but validation of ligands remains problematic because of their infinitely variable chemical character, as opposed to the very limited repertoire of the standard residue types (Kleywegt, 2000). As a result, the typical quality of the ligands is considerably poorer than that of the macromolecules, for which refinement dictionaries generated by experts are readily available (Davis et al., 2003, 2008; Kleywegt, 2007).

The binding pose of the ligand should make sense in terms of the H-bonding, salt-bridge, hydrophobic, and ring stacking interactions with the surrounding chemical groups, including protein or nucleic acid atoms, and metals and other ions, other ligands, and solvent molecules. However, no tools, apart from clashes, are currently available to evaluate these interactions.

A high-quality description of the geometry and stereochemistry of every new ligand (bond lengths and angles, planar groups, chiral centers, etc.) is needed, preferably derived by analysis of accurate small-molecule crystal structures (Engh and Huber, 1991). At present, such descriptions are not generally available but could be derived for most ligands using a tool such as Mogul (Bruno et al., 2004). Mogul analyzes geometrical parameters (bond lengths, angles, torsions) by mining structures from the Cambridge Structure Database (CSD; Allen, 2002) to produce the distribution of each parameter as observed in small-molecule crystal structures containing similar fragments. Thus, if a sufficiently large number of examples of the parameters involving the same atom types can be found in the database, the average and standard deviation of a parameter's distribution can be calculated, assuming a unimodal and approximately normal distribution, and from this the Z-score of the parameter value observed in the deposited crystal structure.

Ligand geometry can be optimized with quantum-chemical calculations, as in eLBOW (Moriarty et al., 2009) or by molecular mechanics calculations, as in the PRODRG server (Schüttelkopf and van Aalten, 2004). Yet another alternative is to compare the geometry of a ligand with any other instances of that same ligand in the PDB, an approach taken in ValLigURL (Kleywegt and Harris, 2007). wwPDB deposition sites currently use the ideal geometry, stereo-chemistry, and standard names defined in the chemical components dictionary (Henrick et al., 2008) when ligands are deposited that already occur in the PDB.

The Cambridge Crystallographic Data Centre (CCDC) has recently entered into collaboration with the wwPDB partners. As part of the agreement, wwPDB will have access to Mogul, which will be integrated into the wwPDB validation pipeline, and to the experimental coordinates of ligands that are or have been deposited in the PDB and that also occur in the CSD. Taken together, this means that high-resolution reference coordinates will become available for many ligands in the PDB and that high-quality geometry-validation reports can be generated for all ligands that will be deposited in the future.

Fit to electron density can be calculated as an absolute number such as the RSR value or RSCC, but for all but the most common ligands it will be impossible to obtain a statistically significant sample of instances of that ligand for comparison purposes. Thus, statistics such as RSR Z-scores cannot be calculated for most ligands (Kleywegt et al., 2004). Another problem is that ligands can be quite large, so scores that treat them as single residues can be insensitive to local density fit. Nonetheless, a comparison of the raw RSR score with that of the protein will give a clear indication of whether the ligand fits the density as well as the protein. For instance, a ligand with an RSR score that is double the average for the protein should almost certainly be inspected.

The VTF recommends that ligands with geometrical parameters judged by Mogul to be outliers should be flagged as concerns, as should ligands for which the RSR value is more than double the average value for the protein component. This threshold could be revised later in light of statistical analysis of RSR values for ligands in the PDB.

#### Ions and Other Solvent Components

Even at high resolution, some solvent components are nearly iso-electronic and thus are difficult to distinguish based purely on electron density. At lower resolution, distinguishing components of similar shape becomes even more difficult. Other information must be invoked, such as strength of anomalous scattering, interatomic distances, and coordination geometry. When this is done, it is clear that some components have been misidentified; for example, a study of metal coordination geometry strongly suggests that metal ions have been misidentified in a number of PDB entries (Zheng et al., 2008).

Although there have been many studies on the preferred environments of different ions (e.g., Harding, 2006), we are not aware of any convenient tools for validating ions and other solvent components. When such tools are available, they should be incorporated in the validation pipeline at the wwPDB sites.

### Incomplete Models

A complication arises when incomplete models are deposited. For instance, a very low-resolution study may allow for only a backbone tracing, leading to a deposited model consisting of only Cα atoms with or without assigned sequence. Similarly, unknown ligands may be modeled as a set of “unknown” atoms. Clearly, validation of such models is complicated. For Cα-only protein models, a few basic geometric validation criteria have been described (Kleywegt, 1997) and they can be used to detect grossly mistraced models. Depending on how the model was built and refined, it may in some cases also be possible to investigate criteria such as the radial distribution of B-factors or the compatibility of sequence and fold (Bowie et al., 1991). If multiple copies of the molecule are present in the asymmetric unit, NCS-based statistics and plots can be inspected. If related structures are available from the PDB, they could be compared with the new model, for example, to detect possible register errors. For models of “unknown” ligands, little can be done other than checking the fit of the model to the density if atoms are of known chemical type and possibly checking unfavorable contacts with properly modeled entities such as protein.

### The Role of Re-refinement

With the mandatory deposition of structure factors, it is now possible to re-refine all newly deposited crystal structures, which has been done for the PDB_REDO databank of re-refined PDB entries (Joosten and Vriend, 2007). Re-refinement is, of course, not the task of the PDB, but its results have implications for validation.

The most trivial is data integrity checking. The PDB_REDO evaluation of PDB entries resulted in roughly 400 reports of formatting errors in PDB entries that were corrected by PDB annotators. Some of these errors (*e.g.*, wrong atom names, missing R-values, erroneous MTRIX records, improperly annotated reflection data) can cause false results in the validation methods described above. In addition, the re-refinement exercise highlights any lack of ancillary information (e.g., restraint libraries, specifications of NCS or TLS [translation/libration/screw] groups) needed to carry out a similar refinement or for some aspects of validation. Perhaps of greater importance, the user is not able to judge the significance of deviations from expected geometry, particularly for ligands, without knowledge of restraints, of variation among B-factors without knowledge of TLS groups, or of variation among NCS copies without knowledge of the NCS groups.

The crystallographic residual R could not be reproduced within 0.05 for up to 10% of all PDB entries with experimental data when the computation is carried out with the Electron Density Server (EDS; Kleywegt et al., 2004) or PDB_REDO. For R_free_, the failure rate for reproducing deposited values is significantly higher, generally because the necessary R_free_ flags were not properly deposited. The VTF recommends validation of the R_free_ test set upon deposition.

The re-refinement of a large subset of the PDB has shown that for many PDB entries the geometric validation scores can be improved by optimizing basic refinement parameters without explicitly refitting atoms in the structure models (Joosten et al., 2009).

### Presentation of Results for Depositors and Users

The depositor and user communities require information from many of the same validation tools, but they have very different requirements for how that information is organized and presented. The depositor needs both global and detailed presentation of potential problems highlighted by a large array of validation tools, preferably ranked by severity. Depositors, referees, and some other users need a comparison with the group of PDB structures at similar resolution to judge how well the model made use of the experimental data (note that these comparisons will change somewhat over time, particularly for low-resolution structures, as methods improve).

A “test” validation pathway at the PDB deposition site, including all steps performed on deposited structures, would help depositors to correct errors before actual deposition. This would require that the validation of entries be fully automated, i.e., free of human intervention by PDB staff. To facilitate correction of local errors in a structure, individual-residue results should be presented in machine-readable format for export to display programs (see below).

**Figure 6:**
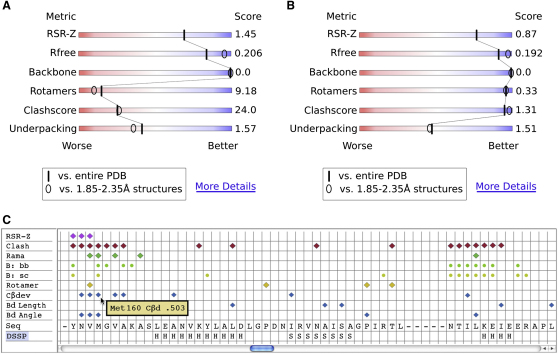
Possible Representations of Validation Metrics (A and B) Slider representation of key validation metrics for the verotoxin-1 B-subunit (Stein et al., 1992) before (A) (PDB entry 1bov) and after (B) (PDB entry 2xsc) a rebuild and rerefinement (Robert D. Oeffner & Gábor Bunkóczi, personal communication). The color scale across each bar represents the percentile score for each metric, with better scores to the right in blue. The solid bars show percentile relative to the entire PDB, whereas the ellipses show percentile relative to structures at similar resolution (2.1Å here). Note that the RSR-Z score is defined only on the all-PDB scale. This structure predates introduction of R_free_ (Brünger, 1992); the value reported here was obtained by 10 macrocycles of refinement in phenix.refine (Afonine et al., 2005), after applying a 0.5Å random shift to all atoms. (C) Displaying per-residue validation on a scrollable plot. Outliers are flagged for real-space residual, all-atom clashes, conformation, and covalent-geometry, along with sequence and secondary-structure assignment. One-letter code enables a concise view, with further details shown on mouse-over. This example is from an entry at 2.7Å resolution, with average problems in the core but misfit regions at several transitions between helix and disordered loop. Plot modified from output of the MolProbityCompare utility by Bradley Hintze.

The user, in contrast, needs first an indication of absolute global quality, to make good choices among similar structures, and then needs easy access to information about local quality to judge the reliability of inferences that depend on specific atoms or residues. This information should be easily understandable by scientists from many disciplines and should not require a deep understanding of crystallographic or validation methodology. Each criterion should clearly measure quality, but should not depend strongly on arbitrary cutoffs, because community standards change with time and user needs differ. These goals are achievable by presenting each global validation metric for an individual structure relative to the distribution of that metric across the entire PDB. The possibility favored by the VTF is to use a percentile score, defined as the percentage of structures in the PDB (or in the resolution range) with a poorer score than the structure under consideration. Figures 6A and 6B shows a possible graphic representation of such percentile scores. The VTF recommends that a summary of overall structure quality be shown on the main page of each PDB entry; the suggested key criteria are listed in Table 1, in which the “ideal values” are those achieved for low-B regions of very high-resolution structures. It would be helpful to provide links to explanatory documentation, detailed local and global criteria, and depositor comments.

Although many validation criteria can be presented as points on a distribution, there are some yes/no criteria better given as potential “concerns” or “unusual features.” This has the advantage that results of the corresponding tests need only be presented when worse than some threshold, thus reducing information overload. The term concern would cover serious potential errors that could be addressed by the depositor, such as errors in cell constants or wavelength, misassigned symmetry, or extreme geometry problems. “Concerns” would also flag annotations for issues such as Cα-only coordinates, unknown sequence, or an unknown ligand. Because these concerns are not necessarily errors, depositors should be allowed to comment on them, and their comments should be linked to the flags. For example, crystals can possess pseudosymmetry, which can be demonstrated by careful analysis beyond the current capabilities of automation. Table 2A presents a list of criteria that would give rise to “concerns.”

The term unusual feature would cover features of the model or data that are not under depositor control but may still have an impact on the quality or reliability of the structure, such as the presence of twinning, translational noncrystallographic symmetry, severe anisotropy, or unusual or challenging experimental conditions. Alternatively, a PDB entry could result from the test of a new method, with a deliberate choice made not to optimize certain validation criteria. Possible unusual features are open-ended and may often be identified by the depositor rather than by validation. Table 2B shows examples of criteria that would give rise to “unusual feature” flags.

### Presentation of Results for Referees

Only a tiny proportion of PDB entries contain structures with catastrophic errors, but the consequences of these few structures can be severe, both by misleading researchers who build on the structural results and by reducing confidence in structural biology in general. Fewer such errors would enter the literature if referees were given access to data that would allow them to evaluate the validity of the structural claims made in the manuscript or to request improvements in the overall quality of the structure.

We propose a simple means to enhance the information available to referees, inspired by a suggestion made by George Sheldrick on the CCP4 bulletin board (August 18, 2007). When the deposition is complete and a PDB code assigned, the depositor would be sent a validation summary suitable for use by a referee, reporting quality indicators that are widely accepted and understood within the structural biology community. With current technology, a useful and accessible format for the report would be a PDF. The first page would give an overall summary, with key percentile scores for global quality on both all-PDB and resolution-relative scales. The first page would also present any “concerns” or “unusual features” present in the structure or data and give per-chain quality indicators, including mean B-factor, overall RSR-Z, and overall RMS-Z for bonds, angles, and planes. Subsequent pages would provide detailed information on residue-based quality indicators, allowing the referee to assess the level of confidence for specific residues discussed in the manuscript. They might be presented either as lists of outliers or as a multicriterion plot along the sequence. In either case, outlier thresholds should be adjusted to avoid information overload. The suggested contents of a summary referee report are listed in Table 3.

In the short term, referees would know that such a summary report is available and should request it through the editors before agreeing to referee the paper. In the longer term, we encourage journals to require authors of structural papers to supply the summary report together with the manuscript. To provide confidentiality, the VTF recommends that it should be possible to delay the appearance of any information about the deposition on wwPDB public databases (including the mere fact of deposition) until the author approves release of the entry or the publication appears in print, whichever comes first.

### Detailed Validation Results

Depositors and expert users will need access to further detail on the validation results, which should be available both on the validation report provided to the depositor and to the users, though details need not be prominently displayed. Table 4 summarizes these remaining validation results, as recommended in this report.

### Exporting Global and Local Quality Information

The complete data underlying both global and local validation reports (outlined in the tables) should be available for download or from a web service in a simple machine-readable format that is easily parsed by clients (software receiving wwPDB data and annotation). The exact details of the format need to be resolved in consultation with authors of the client applications. The key criteria, concerns, and per-chain scores should support reproduction of the PDB web page summary or referee report summary. The local per-residue scores should support the production of outlier lists at chosen thresholds or of scrollable, multicriterion displays along the sequence. One possible form of such a display is shown in Figure 6C. The validation data should preferably be separate from the coordinate file, because the bulk of its organization is by residue rather than by atom, and its detailed content is expected to evolve.

### Additional Recommendations to the wwPDB

Additional recommendations that were made to the wwPDB by the Validation Task Force, about the presentation of results and practical issues of validation, are listed in the Supplemental Information.

## Figures and Tables

**Table 1:** Key Validation Criteria

Validation criterion	Ideal score	Median for 1.5/3Å structures
R_free_	Undefined	0.21/0.28
Real-space residual (% RSR-Z > 2)	Undefined	2.7 (resolution independent)
Clashscore (clashes per 1000 atoms, including H)	<5	8.8/39
Under-packing	1	1.2/2.2
Ramachandran score (% outliers)	0.05	0/1.7
Rotamer score (% poor)	0.5	1.7/9.6
Buried H-bonds (fraction unsatisfied)	0.02	0.025/0.08
RNA ribose puckers (% poor)	0.5	0/2.7

**Table 2A:** Concerns and Unusual Features: Criteria to Flag “Concerns”

Validation criterion	Threshold
Poor overall geometry	Percentile <0.1 for RMS-Z score of bonds, angles, or planes, or percentile <0.1 for fraction outliers of bonds, angles, or planes
Extreme local geometry	Any individual outliers >15σ
Inverted chirality	Incorrect chirality at a Cα (or for a nucleic acid sugar)
Intensity outliers	>10% of reflections have a Wilson probability lower than 10^−6^
Data labels	Structure amplitudes (F) labeled as intensities (I) or vice versa
Symmetry	Data analysis shows missed symmetry within experimental error but no evidence of twinning, except if depositor indicates that using NCS symmetry produces significantly lower R_free_.
Twinning	Estimated twin fraction >0.2, but no indication that a twin target was used for structure refinement
Translational NCS	Nonorigin peak in native Patterson >75% of origin, and position of peak is consistent with missed centering operator
Incomplete structure	Cα-only coordinates, or no sequence information
Ligand problems	RSR > twice average for protein, poor stereochemistry
Carbohydrates	Nomenclature is inconsistent with structure, or N-glycan chain contains residues that are not known to occur naturally

**Table 2B:** Concerns and Unusual Features: Examples of “Unusual Features”

Twinning	L-test indicates a twin fraction >5%
Severe anisotropy	ΔB/B_mean_ > 1
Translational NCS	Nonorigin peak in native Patterson >75% of origin, but peak position not consistent with missed centering operator
New or unusual methods	For example, ensemble or free-atom refinement
Unusual experimental conditions	For example, extreme pH or pressure cell

**Table 3:** Content for Referee Summary Report

Section	Information presented
Global summary	Scores and percentiles for key criteria
	Concerns, unusual features, and depositor comments
	Per-chain quality indicators: mean B-factors, mean RSR-Z, and overall RMS-Z for bonds, angles, and planes
Per-residue flags	RSR-Z score >2
	Bond, angle, and planarity outliers with absolute Z-score >5
	Ramachandran outliers <0.05% probability
	Residues with clash overlap >0.5Å
	Ribose pucker and backbone conformation outliers in RNA

**Table 4:** Additional Detailed Criteria for Depositor Report and Validation File Export

Validation criterion	Information presented
**Per-residue Data**	

Density fit	RSR-Z score; mean B-factor
Backbone conformation	Ramachandran angles (outliers flagged); secondary structure assignment
Side-chain conformation	Rotamer name and probability; N/Q/H flip score
Geometry	Bond, angle and planarity Z-scores
All-atom clashes	Worst outlier in residue with overlap >0.4Å (more stringent)
Unsatisfied H-bonds	Unsatisfied buried H-bond donor or acceptor
RNA conformation	Ribose pucker outliers; backbone conformer name and score

**Other Plots**	

Backbone conformation	Ramachandran plots (for all 6 categories)
Structure factors	Wilson plot, resolution-dependent completeness
Carbohydrates	CARP plots

## Supplemental Information

Document S1. Three Figures, One Table, and Supplemental Experimental Procedures
