# Supplementary material for: A New Generation of Crystallographic Validation Tools for the Protein Data Bank
Source: Structure. 2011 Oct 12;19(10):1395–412. doi: 10.1016/j.str.2011.08.006 (PMC3195755; doi:10.1016/j.str.2011.08.006)
Supplement: Document S1. Three Figures, One Table, and Supplemental Experimental Procedures [file mmc1.pdf]

Structure *19*

Supplemental Information

A New Generation of Crystallographic Validation Tools for the Protein Data Bank

Randy J. Read, Paul D. Adams, W. Bryan Arendall III, Axel T. Brunger, Paul Emsley, Robbie P. Joosten, Gerard J. Kleywegt, Eugene B. Krissinel, Thomas Lütteke, Zbyszek Otwinowski, Anastassis Perrakis, Jane S. Richardson, William H. Sheffler, Janet L. Smith, Ian J. Tickle, Gert Vriend, and Peter H. Zwart

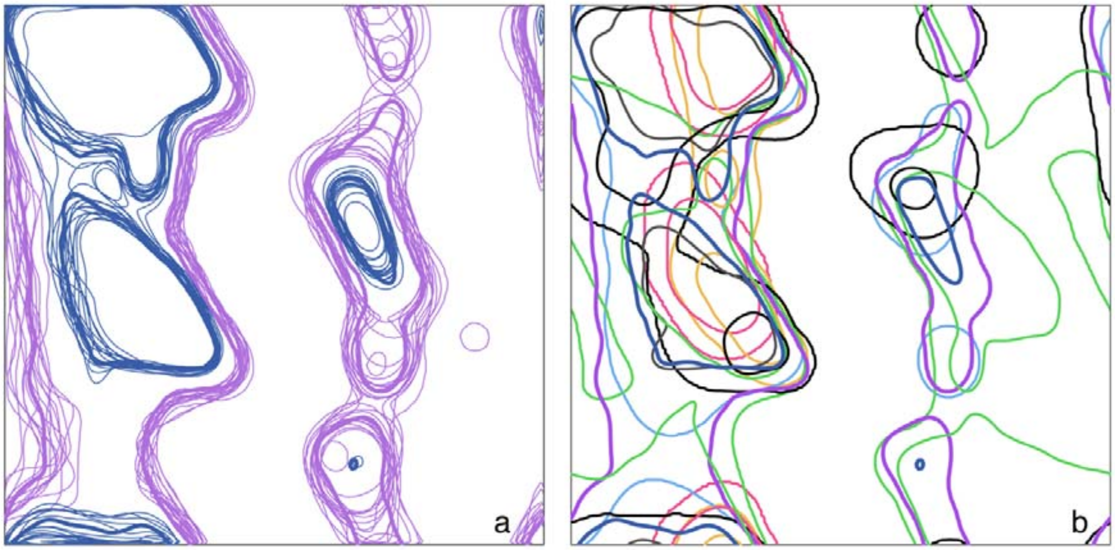

Figure S1, related to Figure 2. Outer contours of Ramachandran plots for specific amino acid categories; in both panels, the general-case contours are shown as wider lines (dark blue and purple). (a) Overlapped contours for each of the 16 amino acid types that are included in the "general" distribution (see Fig. 2B) because they match quite well; 98% contours are in dark blue, 99.95% contours in purple. (b) Overlapped contours for the 6 categories recommended by the VTF (Gly in green, *trans*-Pro in gold, *cis*-Pro in red, pre-Pro in black, Ile/Val in cyan, and general in wider dark blue and purple), proposed for separate evaluation because they are each very different.

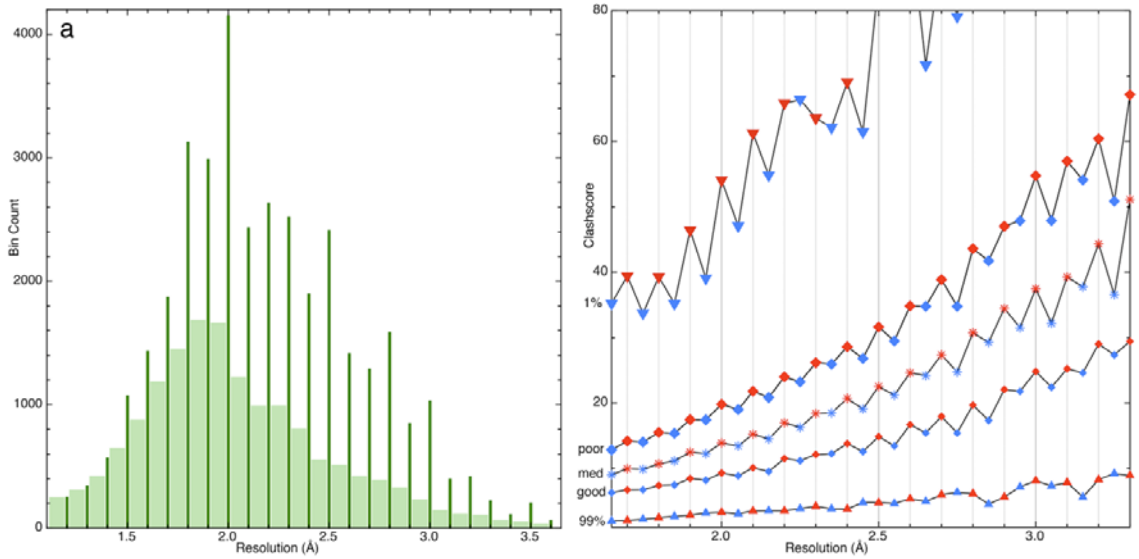

Figure S2, related to Figure 3. Roundoff artefact, for reported resolution values. (a) Count of number of PDB entries reporting exact tenth Å resolution (narrow, dark bars; 20-fold over-represented) and the sum of those reporting each in-between values (wide, light bars). (b) Median, quartile, and extreme percentile clashscore values, for non-overlapping bins covering exact tenth Å resolutions only (red) and in-between resolutions (blue). Entries reporting exact tenth Å resolution values score consistently somewhat worse (higher clashscores).

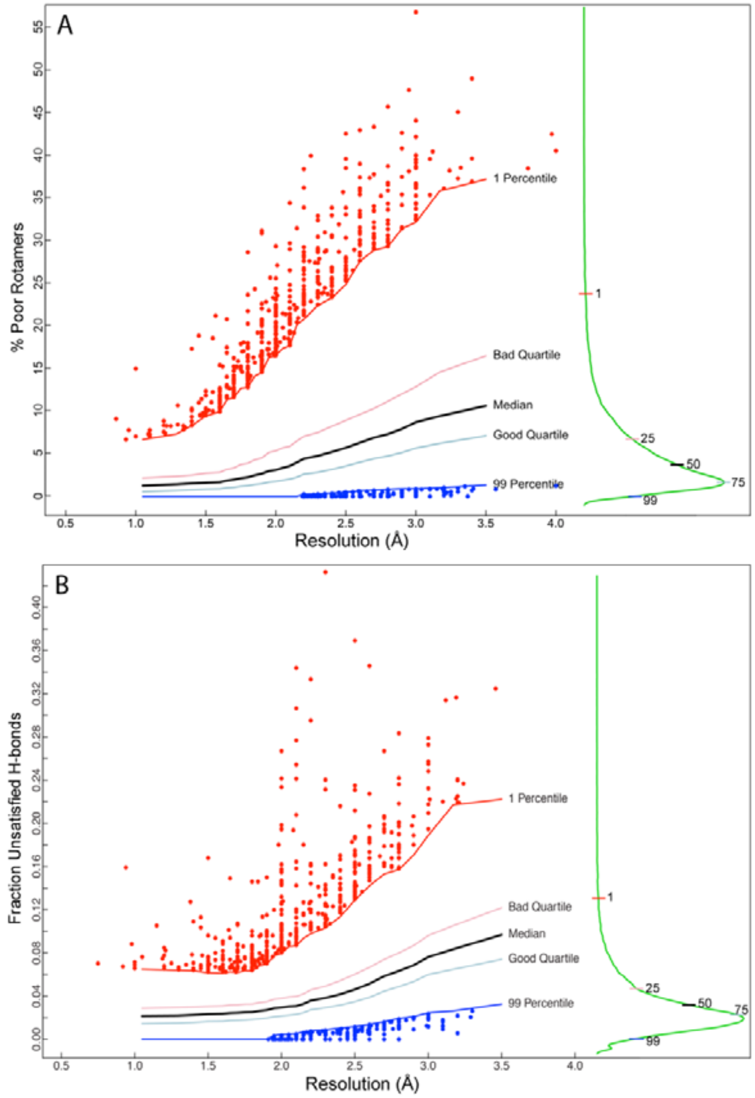

Figure S3, related to Figure 3. All-PDB (X-ray, since 1990) distribution of validation criteria as a function of resolution. Median and quartile levels are plotted smoothly, along with all individual data points for outlier structures beyond the 1st percentile (poor; red) or the 99th percentile (good; blue) values. (See supplementary material for detailed criteria, and for procedures and discussion of these shingle-smoothed, quartile-and-outer-percentile plots with outlier datapoints. At the right of each panel is the resolution-independent, one-dimensional distribution (green line) with median, quartile, and outer percentile values marked, for the aggregated set of all PDB entries. (A) Percent poor rotamers. (B) Fraction of buried hydrogen bond donors or acceptors that are unsatisfied.

Table S1, related to Figure 3.
Defining values for 27 shingle-overlapped bins of resolution (Å).

Bin #	Bin center	Bin_min	Bin_max	count
1	1.03	0.501	1.151	869
2	1.26	0.501	1.451	3273
3	1.40	1.151	1.551	4320
4	1.50	1.351	1.601	4751
5	1.58	1.451	1.699	4807
6	1.65	1.551	1.701	4768
7	1.70	1.601	1.799	4517
8	1.76	1.699	1.801	6466
9	1.80	1.701	1.899	6278
10	1.85	1.799	1.901	7818
11	1.90	1.801	1.999	6349
12	1.95	1.899	2.001	8823
13	2.00	1.901	2.099	7059
14	2.05	1.999	2.101	7835
15	2.10	2.001	2.199	4665
16	2.15	2.099	2.201	6072
17	2.23	2.101	2.301	7148
18	2.30	2.199	2.451	9269
19	2.40	2.201	2.551	9571
20	2.50	2.301	2.651	7943
21	2.60	2.451	2.751	6542
22	2.70	2.551	2.851	5521
23	2.80	2.651	2.951	4729
24	2.90	2.751	3.051	4231
25	3.00	2.851	3.251	3371
26	3.20	2.951	4.001	3307
27	3.50	3.251	4.001	1034

Additional recommendations to the Worldwide Protein Data Bank

As requested by wwPDB, the X-ray VTF has made recommendations about the components and product of the validation pipeline that will be a part of the new deposition and annotation tool currently being developed by the wwPDB partner sites. In addition, the VTF would like to make the following, related recommendations:

- On wwPDB web sites, the front page for any PDB entry should provide users with an intuitive indication of the global quality of the entry by the key criteria.
- Depositors should be urged to include enough information to reproduce the refinement using the deposited coordinates and structure factors. With present technology, this would include cross-validation flags, non-crystallographic symmetry (the definitions of the atoms related by NCS and the target RMSDs), wavelength(s) of data collection, identification of the restraint library and any extra restraints, solvent model, model for atomic displacement parameters (including, if appropriate, TLS parameters and anisotropic U-values), H atom model (if refined but not deposited), identification of refinement target, twinning status and (if relevant) description of twinning. As techniques advance, other information may be required.
- The validation process should be automated as much as possible, so that depositors can freely upload revised coordinates for validation, without increasing the workload of the core PDB staff. There should be a clear "test pathway" for validation, in which structures can be validated outside of the deposition pathway. Data submitted to the test pathway should be deleted upon completion of the validation computations.
- Both global and per-residue validation data should be provided on the wwPDB web sites in a machine-readable format, which will allow users to compare overall quality of related structures and to view annotations of local quality criteria in the context of either sequence or structure, using compliant molecular display programs. Figure 6C shows a possible representation of per-residue validation data as a scrollable plot.
- The validation criteria, including algorithms and cutoff values, should be reviewed regularly by a successor to the current Validation Task Force. We suggest that a five-year cycle would be sufficient to keep up with advances in understanding of structure and validation methodology.

Experimental Procedures

The primary validation criteria were chosen to cover the complementary aspects of experimental data, model-to-data match, geometry, conformation, and packing quality. Preference was given to criteria with a history of broad application, and it was required that freely usable, well documented software for their calculation be available. Each of the key criteria was calculated for all relevant PDB x-ray structures, and the extreme outliers were examined to ensure that they generally identified real problems. In several cases, this process resulted in removal or replacement of bad outlier entries. Since a disproportionate number of the outlier entries are early depositions (< 1% of the entries, but > 30% of the worst outliers on each criterion), the final reference sets of data are here taken only from 1990 onward. Other filters apply for some criteria, such as a minimum size for underpacking and protein-only for unsatisfied H-bonds or Ramachandran outliers. The all-PDB datasets used are very similar but differ slightly between the key validation criteria since they were done by different people; they vary from about 47,000 structures for R_{free} to about 52,000 structures for clashscore. Most measures of model accuracy correlate strongly with resolution, but RSR-

Z scores are already normalized to be resolution-independent. Bond-length outliers show a flat distribution, and bond-angle outliers are only slightly correlated with resolution.

The RMS-Z score is defined as the root-mean-square value of the Z-scores for a particular criterion; the Z-score, in turn, is defined as the deviation from the mean or expected value, divided by the estimated standard deviation. The RMS-Z score is thus a dimensionless quantity, calibrated to reflect the amount of variation expected in each validation measure. Typically, the Z-score is computed using the population standard deviation, where the population can be the entire PDB, structures at similar resolution or (in the case of bond lengths, bond angles and planarities) the set of small molecule structures examined by Engh and Huber (1991; 2001).

$$Z = \frac{x - \langle x \rangle}{\sigma(x)}$$

$$\text{RMS-Z} = \sqrt{\tfrac{1}{n}\sum_{i=1}^{n} Z_i^2}$$

Most validation criteria can be satisfied more easily as the resolution of the diffraction data increases, so that the mean values of the criteria vary significantly with resolution. In order to evaluate the quality of a structure relative to what could be expected for the available data, it is necessary to account for the influence of resolution, most readily by comparison with a set of structures determined at similar resolution. There is a trade-off between choosing a sufficient number of structures for comparison, to reduce statistical error, and choosing too wide a range of resolution, over which there would be real variation. In a previous study, we found that as few as 400 structures at similar resolution could be used to compute the mean and standard deviation of validation criteria (Read and Kleywegt, 2009); at most resolution limits, this requires only a narrow range of resolutions. However, some smoothing is required to avoid the resolution roundoff artefacts documented in Figure S2.

Selecting cutoff values for scoring or listing outliers is not an exact process, but the cutoffs have a strong influence on the usefulness of validation reports. The cutoffs recommended here were guided by validator and user experience with each individual measure. The optimal cutoff value should flag a large fraction of the real problems, but including a significant number of false positives is counterproductive. The cutoffs should be reassessed periodically to balance those two criteria and may need to vary with resolution or molecule type.

Categories for Ramachandran validation

Categories for Ramachandran validation were chosen according to which amino acids had very similar (Figure S1a) or very different (Figure S1b) contours at the 98% and 99.95% levels used for validation. These distributions were made from a MySQL (MySQL, 2006) database containing PDB and validation data for over a million residues from 4400 non-homologous chains (at the 70% sequence identity level), chosen for resolution < 2.0Å and an average of resolution and MolProbity score (Chen *et al.*, 2010) of < 2.0. Individual residues were omitted if they had occupancy < 1.0 or any backbone B-factor > 30. Contours were produced as kernel plots with density-dependent smoothing (Lovell *et al.*, 2003); a contour described as 99.95% means that 99.95% of the filtered data is enclosed by that contour. (Data analysis and kinemage graphics for Figures 2 and S1 by Daniel Keedy.) To evaluate an individual residue for validation, the appropriate distribution is chosen by a priority heirarchy

of Gly,Pro > pre-Pro > Ile/Val > general (e.g., a Gly that is also a pre-Pro is judged on the Gly distribution, which is the more unusual). That 2D distribution of values (on a 2° grid of ϕ □□□ ψ) is interpolated to give the score, which counts as a Ramachandran outlier if it is > 99.95 (1 in 2000). An analogous procedure is followed for side-chain rotamers, but in the relevant number of dimensions from 1 to 4. The current dataset for rotamers is older, as well as including more divisions and more dimensions, and poor rotamers are flagged only at the 1 in 100 level; future compilations should be able to do better than that.

Shingle-smoothed quartile and outer-percentile plots with outlier datapoints

Producing all-PDB plots of the various validation criteria with smoothed lines for percentile boundaries and individual datapoints for the extreme 1% outliers was more difficult than one would expect. It is immediately evident that score *vs*. resolution dependencies are not linear (Figures 3, 4 and S3). Quadratic or log-scale fits are not appropriate either: some criteria plateau at high resolutions, while others have a high occurrence of good entries with genuinely zero outliers. The dispersion (vertical distributions) at specific resolutions cannot be modeled by common probability distributions. Many of the validation criteria have a lower bound for good values and a long tail for large outliers, with a shape that does not fit even a Poisson distribution. For such cases, median-based statistics are more appropriate than mean and standard deviation (such as used for "box and whisker plots"), so the all-PDB distributions are analyzed and reported as percentile scores.

Resolution is clearly the most robust and meaningful measure for the information content of diffraction data, but it is not a precise measure because of both technical and personal-preference differences in exactly how resolution is defined and in how much that value is rounded off. Initial attempts to plot smooth percentile lines from non-overlapping bins of resolution encountered a surprising artefact from this imprecision of definition and rounding: quite consistently for most validation criteria, entries reporting exact tenths of Å for resolution score somewhat worse than entries reporting in-between values (see Figure S2b for clashscore). PDB headers report only two decimal places for resolution, so in the absence of rounding the ratio of exact tenths to in-between values should be only 1:9; in fact the ratio is about 2:1, as shown by the bin counts in Figure S2a. Factoring in year of deposition reduces the discrepancy only by 1/3 to 1/2; the rest is presumably caused by some combination of the tendency to round toward better resolutions, and perhaps that those who report both precise and conservative values also tend to take more care in other ways. Since these factors do not correctly represent the inherent influence of data quality on structure quality, the reference percentile plots for validation criteria should be more suitably smoothed. For the plots shown in this paper, a set of "shingle-overlapped" (in progressive sets of 3) resolution bins was defined (Table S1), producing much smoother lines: compare the quartile lines in Figure 3B with the jagged versions in Figure S2b. In the main text figures, data points for individual entries are shown only outside of the poor 1 percentile line (in red) and the good 99 percentile line (in blue), since the all-PDB distributions are completely saturated toward the center. The resolution-dependent, one-dimensional distribution is shown at one side as a green line with the median, quartile, and extreme-percentile values marked (note that the median is always well above the modal value in these highly skewed distributions). A script to produce these plots in the R statistics program (Team RDC, 2005) was developed jointly by WBA, WS, and JSR and used for Figures 3, 4 and S3; it is available from the web site at http://kinemage.biochem.duke.edu, under “Software”. Figure S3 shows percentile plots for rotamer outliers and unsatisfied buried H-bond donors/acceptors, to complete the basic data distributions for all key metrics.

References

Chen, V.B., Arendall, W.B. III, Headd, J.J., Keedy, D.A., Immormino, R.M., Kapral, G.J., Murray, L,W., Richardson, J.S., and Richardson, D.C. (2010). "MolProbity: all-atom structure validation for macromolecular crystallography", Acta Cryst. D*66*, 12-21.

Engh, R.A. and Huber, R. (1991). Accurate bond and angle parameters for X-ray protein structure refinement. Acta Cryst. A*47*, 392-400.

Engh, R.A. and Huber, R. (2001). Structure quality and target parameters. In International Tables for Crystallography Vol. F, M.G. Rossmann and E. Arnold, eds. (Dordrecht, The Netherlands: Kluwer Academic Publishers), pp. 382-392.

Lovell, S.C., Davis, I.W., Arendall, W.B. III, de Bakker, P.I.W., Word, J.M., Prisant, M.G., Richardson, J.S. and Richardson, D.C. (2003). Structure validation by Cα geometry: ϕ, ψ and Cβ deviation. Proteins *50*, 437-450.

MySQL AB. (2006). MySQL administrator's guide and language reference, 2nd ed. Indianapolis, IN: MySQL Press.

Read, R.J. and Kleywegt, G.J. (2009). Case-controlled structure validation. Acta Cryst. D*65*, 140-147.

Team RDC. (2005). R: a language and environment for statistical computing. Vienna, Austria: R Foundation for Statistical Computing.
